# Influence of Early Multidisciplinary Collaboration on Prevention of ICU-Acquired Weakness in Critically Ill Patients

**DOI:** 10.1155/2022/3823368

**Published:** 2022-07-30

**Authors:** Bolan Wang, Xiqiang He, Shujun Tian, Can Feng, Wenbin Feng, Limin Song

**Affiliations:** ^1^Department of Nursing, Xiangtan Central Hospital, Xiangtan, 411100 Hunan, China; ^2^Department of SICU, Xiangtan Central Hospital, Xiangtan, 411100 Hunan, China; ^3^Department of EICU, Xiangtan Central Hospital, Xiangtan, 411100 Hunan, China; ^4^Department of Rehabilitation, Xiangtan Central Hospital, Xiangtan, 411100 Hunan, China

## Abstract

**Objective:**

This study focused on elucidating the influence of early multidisciplinary collaboration on preventing intensive care unit- (ICU-) acquired weakness (AW) in critically ill patients (CIPs).

**Methods:**

Ninety-five CIPs admitted between December 2018 and December 2021 were selected and assigned to the following two groups according to the intervention pattern: the control group (the Con; *n* = 40) treated with routine early rehabilitation intervention, and the research group (the Res; *n* = 55) intervened by early multidisciplinary collaborative intervention. The incidence of complications (ICU-AW, deep vein thrombosis (DVT), and pressure ulcers (PSs)) and recovery indices (days of ventilator use, ICU treatment time, and length of hospital stay (LOS)) were recorded. Besides, patients' activity function and quality of life (QoL) were evaluated and compared, among which the former was evaluated by the Barthel Index (BI), ICU Mobility Scale (IMS), and Medical Research Council (MRC) Scale, and the latter was assessed by the World Health Organization Quality of Life Assessment (100-item version) (WHOQOL-100).

**Results:**

The data identified statistically a lower incidence of complications (ICU-AW, DVT, and PSs) and shorter time of ventilator use, ICU residence, and LOS in the Res compared with the Con. In addition, BI, IMS, MRC, and WHOQOL-100 scores in the Res elevated statistically after treatment and were higher than those of the Con.

**Conclusions:**

Early multidisciplinary collaboration can validly prevent ICU-AW in CIPs, reduce the incidence of DVT and PSs, and promote patients' rehabilitation, mobility, and QoL.

## 1. Introduction

Critically ill patients (CIPs) are often accompanied by multiple organ dysfunction, which not only increases the risk of death but also imposes a certain social burden on medical care [1]. Intensive care unit- (ICU-) acquired weakness (AW), a common acute neuromuscular injury in CIPs, is associated with poor short- and long-term outcomes [2]. It will not only lead to prolonged mechanical ventilation and increased medical costs but also increase ICU treatment time, length of hospital stay (LOS), and even the risk of inpatient-related death [3]. ICU-AW is known to be induced by one or more factors, including severe polyneuropathy, myopathy gravis, long mechanical ventilation duration, and long-term exposure to norepinephrine [4, 5]. The disease generally brings systemic and symmetrical negative effects to patients, mainly affecting the proximal limbs and respiratory muscles, with little impact on the facial and ocular muscles [6]. For CIPs, ICU-AW will affect their rehabilitation process and pose a great threat to their activity function and quality of life (QoL) [7]. According to the relevant epidemiological data, the incidence of ICU-AW is 25-31% in internal medicine ICU and 56-74% in surgical ICU, and patients may also suffer from physical dysfunction, dysfunction, cognitive impairment, depression, and anxiety disorders after discharge [8]. Therefore, an effective intervention model is urgently needed to reduce the occurrence of ICU-AW in CIPs, help them speed up the rehabilitation process, and improve their activity function and QoL.

Early rehabilitation intervention is a series of rehabilitation activities given to patients during critical illness ranging from activity beyond the range of motion to full ambulation [9]. Due to the confirmed effectiveness, reliability, and safety of this intervention in the rehabilitation of CIPs, several countries have issued practice guidelines to guide its implementation [10]. Early multidisciplinary collaboration is also an early rehabilitation intervention model, but its intervention measures are often complex, which is mainly reflected in the need for properly trained interdisciplinary teams to provide early care services [11]. Early multidisciplinary collaboration has a wide range of applications in medical settings such as tuberous sclerosis, breast cancer, and head and neck neoplasms, which can not only promote patient recovery, reduce the risk of adverse events, but also improve the QoL of patients [12–14]. This rehabilitation intervention model has also been applied to ICU patients, which facilitates the recovery of patients' physical function, muscle strength, and autonomous walking function, and reduces the incidence of ICU-AW [15].

This paper explored the application effect of early multidisciplinary cooperation model in CIPs and its influence on the incidence of ICU-AW, patients' recovery, QoL, etc., aiming at providing a new clinical reference for the early rehabilitation of CIPs.

## 2. Data and Methods

### 2.1. General Data

CIPs (*n* = 95) admitted to our hospital during the period of December 2018 to December 2021 were selected and assigned to the following two groups according to the intervention pattern: the control group (the Con; *n* = 40) treated with routine early rehabilitation intervention and the research group (the Res; *n* = 55) intervened by early multidisciplinary collaborative intervention. The Con comprised 25 males and 15 females, with an average age of 62.83 ± 6.77 years. In the Res, the male-to-female ratio and the mean age were 34 : 21 and 61.38 ± 12.32 years, respectively. This study was conducted only after obtaining approval from the Ethics Committee of Xiangtan Central Hospital, and all subjects were aware of the purpose of this research and provided informed consent.

### 2.2. Eligibility Criteria

Inclusion criteria are as follows: CIPs, aged above 18, who were admitted to the ICU for the first time and were eligible for early multidisciplinary collaboration, with clear consciousness, referral to the general ward due to improved condition, life expectancy above 6 months, and stable vital signs were included.

Exclusion criteria are as follows: nervous system diseases or malignant tumors, pregnant or lactating patients, severe neuromuscular diseases, patients with limb disabilities, multiple critical diseases, and severe cardiopulmonary diseases.

### 2.3. Intervention Methods

In routine early rehabilitation intervention model, the responsible nurse instructed the patient to be in a good limb or functional position to prevent spasm, and helped him/her turn over every 2 hours. In addition, the responsible nurse evaluated the patient's consciousness and muscle strength on a daily basis, and gave a full range of passive joint movements with 10 repetitions each and 2-3 cycles of practice per day. Active joint movement was carried out step by step on the basis of passive joint activities. Furthermore, patients were trained in daily activities such as eating and dressing 2-3 times a day. Moreover, exercises guidance such as sitting at the bedside, standing up in a sitting position, stepping in place, and walking were given.

In early multidisciplinary collaborative intervention model, an early multidisciplinary collaborative team was established, with members including attending physicians, rehabilitation therapists, psychological counselors, nutritionists, and nurses. After admission, early rehabilitation intervention programs were formulated according to the disease type, disease severity, nutritional status, psychological status, and social support of patients. Health education was given before rehabilitation intervention. Nurses communicated patiently and in detail with patients to make them understand the necessity and importance of rehabilitation training, so as to help patients build confidence in rehabilitation training. Rehabilitation therapists were responsible for early rehabilitation training of patients, with the training contents the same as the Con. In addition, patients' consciousness and muscle strength were evaluated every day, and the training was conducted in a step-by-step manner to avoid pressure and discomfort. Nutritionists also gave patients nutritional support and developed personalized nutrition programs according to their eating conditions, nutritional indicators, and personal hobbies. For the psychological state of patients, psychological counselors were there to provide psychological counseling for patients via communicating with patients every day to understand their psychological dynamics and demands and providing targeted counseling to reduce their adverse emotions as much as possible, so as to improve patients' treatment compliance. Moreover, nurses paid close attention to the changes of patients' vital signs and conditions, and reported and dealt with patients' maladjustment in a timely manner.

During hospitalization, the Con received routine early rehabilitation intervention, and the Res received early multidisciplinary collaborative intervention.

### 2.4. Outcome Measures

1. Safety. We recorded and analyzed the incidence of complications, including ICU-AW, deep vein thrombosis (DVT), pressure sores (PSs), and ventilator-associated pneumonia (VAP)
2. Recovery indicators. The days of ventilator use, ICU treatment time, and LOS of the subjects were recorded
3. Activity function. We assessed patients' activity function with the Barthel Index (BI; score range: 0-100), ICU Mobility Scale (IMS; score range: 0-10), and Medical Research Council (MRC) Scale (score range: 0-60) [16–18], with the scores of all the three measures in direct proportion to the subjects' ability of daily living, mobility, and muscle strength, respectively
4. QoL. We used the World Health Organization Quality of Life Assessment (100-item version) (WHOQOL-100) [19] to assess the QoL of the research participants from the physiological, mental, and environmental fields as well as the total QoL score. The score (0-100 points) was proportional to the patient's QoL

### 2.5. Statistical Processing

Data statistics and image rendering were performed using GraphPad Prism 6 (GraphPad Software, San Diego, USA). The counting data were represented by number of cases/percentage [*n* (%)], and a chi-square test was employed for intergroup comparisons. The mean ± standard deviation (mean ± SD) was used to indicate the measurement data, and the difference between groups and within groups before and posttreatment was identified by independent samples *t*-test and paired *t*-test, respectively. The threshold of significance was *P* < 0.05.

## 3. Results

### 3.1. General Data

Sex, age, mean age, Acute Physiology and Chronic Health Evaluation (APACHE) II, partial pressure of carbon dioxide (PaCO_2_), partial pressure of oxygen (PaO_2_)/fraction of inspiration O_2_ (FiO_2_), disease type, smoking history, alcoholism history, and residence differed insignificantly between groups (*P* > 0.05) (Table 1).

### 3.2. Effect of Early Multidisciplinary Collaboration on Complications in CIPs

The safety of the two groups of patients was compared to explore the impacts of the two early intervention models on complications in CIPs. The data showed that the cases of ICU-AW, DVT, PSs, and VAP in the Res were 2, 0, 0, and 2, respectively, while the corresponding cases in the Con were 16, 4, 3, and 6, respectively. The incidence rates of the above four complications were significantly lower in the Res compared with the Con (*P* < 0.05) (Table 2).

### 3.3. Influence of Early Multidisciplinary Collaboration on Recovery Indices of CIPs

**Figure 1:**
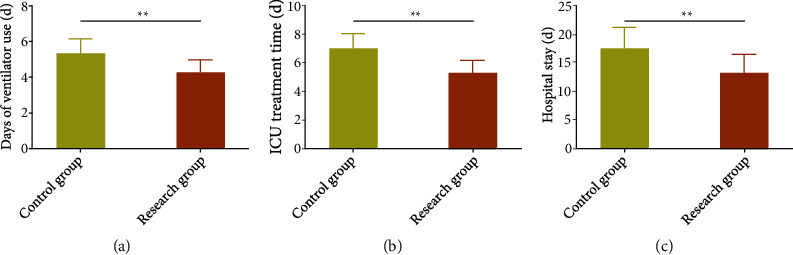
Influence of early multidisciplinary collaboration on recovery indicators of critically ill patients. (a) Influence of early multidisciplinary collaboration on days of ventilator use of critically ill patients. (b) Influence of early multidisciplinary collaboration on ICU treatment time of critically ill patients. (c) Influence of early multidisciplinary collaboration on length of hospital stay of critically ill patients. ^∗∗^*P* < 0.01.

We analyzed the recovery indices to evaluate the impacts of the two intervention models on patient recovery. The data identified notably less ventilator use, ICU treatment time, and LOS in the Res versus the Con (*P* < 0.05) (Figure 1).

### 3.4. Effect of Early Multidisciplinary Collaboration on Activity Function of CIPs

**Figure 2:**
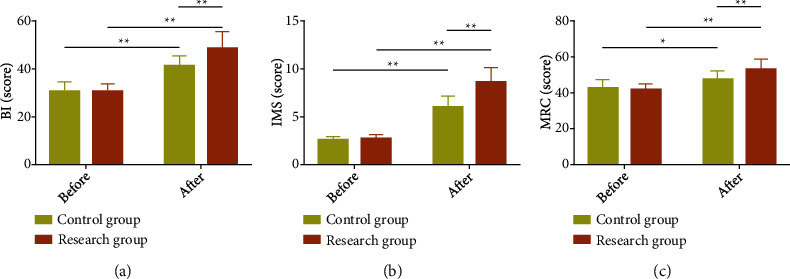
Influence of early multidisciplinary collaboration on activity function of critically ill patients. (a) Influence of early multidisciplinary collaboration on BI in critically ill patients. (b) Influence of early multidisciplinary collaboration on IMS in critically ill patients. (c) Influence of early multidisciplinary collaboration on MRC in critically ill patients. ^∗^*P* < 0.05 and ^∗∗^*P* < 0.01.

Patients' activity function was also compared. After analysis, we found that the activity function showed no statistical difference between groups before treatment (*P* > 0.05), while the activity function of CIPs increased significantly in both groups after treatment (*P* < 0.05), with evidently higher BI, IMS, and MRC scores in the Res compared with the Con (*P* < 0.05) (Figure 2).

### 3.5. Effect of Early Multidisciplinary Collaboration on QoL of CIPs

**Figure 3:**
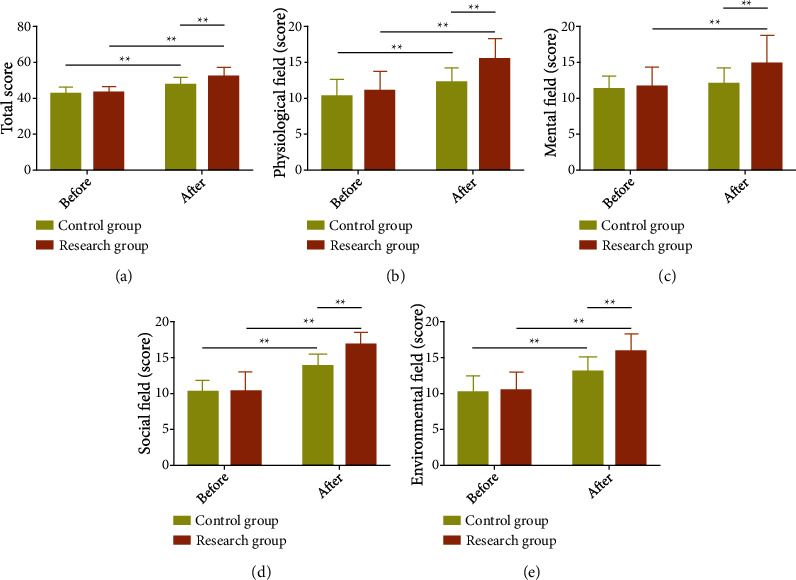
Influence of early multidisciplinary collaboration on quality of life of critically ill patients. (a) Influence of early multidisciplinary collaboration on the total quality of life score of critically ill patients. (b) Influence of early multidisciplinary collaboration on physiological field score of critically ill patients. (c) Influence of early multidisciplinary collaboration on mental field score of critically ill patients. (d) Influence of early multidisciplinary collaboration on social field score of critically ill patients. (e) Influence of early multidisciplinary collaboration on environmental field score of critically ill patients. ^∗∗^*P* < 0.01.

An intergroup comparison was performed on the QoL of CIPs using the WHOQOL-100 scale, so as to analyze the influences of the two interventions on patients' QoL. The data showed no statistical difference in the total score, as well as the scores of various dimensions (physiological field, mental field, social field, and environmental field) between groups prior to treatment (*P* > 0.05). And all the five scores were statistically higher in the Res versus the Con (*P* < 0.05) (Figure 3).

## 4. Discussion

CIPs have a 25-85% risk of developing ICU-AW, with a risk as high as 36% even after discharge [20]. Hence, reducing the incidence of ICU-AW can help promote the early recovery of CIPs, as reported by relevant research [21]. This report reports the effectiveness of early multidisciplinary collaboration model in preventing ICU-AW in CIPs, aiming at contributing to patients' early rehabilitation. We enrolled 95 CIPs and assigned them to two groups according to the intervention model, namely, the Con adopting routine early rehabilitation intervention model and the Res using early multidisciplinary collaborative intervention model. The results are reported as follows.

Besides, ICU-AW, DVT, PSs, and VAP are also common complications in CIPs [22–24]. The reduction of the incidence of these complications can not only improve the QoL and physical function of CIPs but also facilitate their early rehabilitation. More and more researchers are focusing on the prevention and intervention of ICU-AW. For example, Huang et al. [25] pointed out that the combination of mechanical ventilation and pulmonary rehabilitation for ICU patients also had an effective preventive effect on ICU-AW. Wang et al. [26] reported that the application of bundle management strategy in early activities of mechanically ventilated patients not only significantly reduced the risk of ICU-AW but also effectively reduced the incidence of VAP and delirium. And Zhou et al. [27] proposed that early activity combined with early nutritional support for CIPs can help prevent ICU-AW and promote patient recovery. In this study, the incidence of ICU-AW in the Res was 3.64%, significantly lower than that of 40.00% in the Con, suggesting that the early multidisciplinary collaboration model is beneficial to reduce the incidence of ICU-AW. In terms of other complications, significantly fewer cases of DVT, PSs, and VAP were determined in the Res, demonstrating that early multidisciplinary collaboration model can help CIPs prevent these four complications, which is consistent with the report of Zang et al. [28]. The risk factors of ICU-AW in CIPs are known not only related to disease severity, organ failure, and age but also to prolonged immobilization and malnutrition [29]. Under the intervention of early multidisciplinary collaboration, CIPs were provided with rehabilitation training, nutritional support, posture guidance, and psychological counseling based on the specific condition of patients, all of which play a certain role in preventing complications and help patients build confidence in overcoming the disease, contributing to early rehabilitation of CIPs. As for patients' recovery, the Res far outperformed the Con in various recovery indices, which was reflected in shorter duration of ventilator use, ICU treatment time, and LOS, indicating that the early multidisciplinary collaboration model is more conducive to promoting CIPs' early rehabilitation. This model brings together multidisciplinary professionals such as attending physicians, rehabilitation therapists, psychological counselors, nutritionists, and nurses, giving full play to the advantages of multidisciplinary and formulating targeted rehabilitation programs based on patients' individualized conditions. Moreover, the implementation process was based on the principle of gradual progress, which had a positive impact on improving the rehabilitation effect of patients. In the evaluation of activity function, BI, IMS, and MRC in the Res after treatment were significantly higher than those before intervention and the Con, suggesting that the early multidisciplinary collaboration model is more conducive to improving the daily living ability and muscle strength performance of CIPs than the conventional care. This may be attributed to the early rehabilitation training given to CIPs under the early multidisciplinary collaboration intervention model, as well as the daily assessment of patients' consciousness and muscle strength, which allows for a fuller understanding of the body parts of patients with possible mobility impairment, and timely adjustment and better implementation of targeted training in the training process. In addition, the training process based on the basic principle of gradual progress may also have a positive effect on the treatment compliance of patients, so that patients are more cooperative in the rehabilitation training process with more standardized exercises. As reported by Wieczorek et al. [30], the early multidisciplinary collaboration model promotes early mobilization in critically ill children and alleviates movement disorders, similar to our findings. Further, we used the WHOQOL-100 scale to evaluate CIPs' QoL. The Res was also found to have significantly improved QoL that was superior to the Con from physiological, mental, social, and environmental fields, indicating that early multidisciplinary collaboration played a more significant role in improving patients' QoL. Under the early multidisciplinary collaboration intervention model, CIPs have a lower risk of complications with reduced adverse events. Moreover, the activity function and rehabilitation process have been promoted, and the mental state has been well taken care of, which have a positive impact on the QoL of patients. Han et al. [31] also reported that multidisciplinary collaborative continuous nursing significantly improved the QoL of patients with cervical cancer, which was consistent with our research results.

This study has confirmed the effectiveness and safety of early multidisciplinary collaboration model in CIPs and demonstrated its preventive effect against ICU-AW. The main innovation of this study is that it comprehensively evaluates the application value of the early multidisciplinary collaboration model in CIPs from multiple dimensions such as complications, recovery, activity function, and QoL, which provides a comprehensive and effective basis for the management optimization of such patients. However, there are still some deficiencies in this study that need further consideration. First of all, given that this is a small, single-center study, the sample size needs to be expanded to improve the reliability of the results. Second, there is no prognosis research, and supplementing this content will help to clarify the potential impact of early multidisciplinary collaboration model on CIPs' prognosis. Future research will be carried out from the above perspectives to improve this research project.

## 5. Conclusion

Taken together, the application effect of early multidisciplinary collaboration model in CIPs is remarkable, which can prevent ICU-AW and other complications, shorten the rehabilitation process of patients, and improve patients' daily living ability, muscle strength performance, and QoL, which provides an effective intervention strategy for early rehabilitation of CIPs.

## Figures and Tables

**Table 1:** Baseline data of patients in the two groups (*n* (%), mean ± SD).

Factors	*n*	Control group (*n* = 40)	Research group (*n* = 55)	*χ* ^2^/*t*	*P*
Sex				0.005	0.946
Male	59	25 (62.50)	34 (61.82)		
Female	36	15 (37.50)	21 (38.18)		
Age (years old)				0.017	0.895
<60	42	18 (45.00)	24 (43.64)		
≥60	53	22 (55.00)	31 (56.36)		
Average age (years)	95	62.83 ± 6.77	61.38 ± 12.32	0.673	0.502
APACHE II (points)	95	14.52 ± 1.90	15.22 ± 2.24	1.601	0.113
PaCO_2_ (mmHg)	95	49.31 ± 6.37	49.24 ± 7.29	0.049	0.961
PaO_2_/FiO_2_ (mmHg)	95	139.76 ± 6.74	137.11 ± 9.39	1.522	0.132
Disease types				0.918	0.922
Respiratory diseases	32	12 (30.00)	20 (36.36)		
Circulation system diseases	22	11 (27.50)	11 (20.00)		
Digestive system diseases	13	5 (12.50)	8 (14.55)		
Urinary system diseases	14	6 (15.00)	8 (14.55)		
Others	14	6 (15.00)	8 (14.54)		
Smoking history				1.046	0.306
No	39	14 (35.00)	25 (45.45)		
Yes	56	26 (65.00)	30 (54.55)		
History of alcoholism				0.560	0.454
No	35	13 (32.50)	22 (40.00)		
Yes	60	27 (67.50)	33 (60.00)		
Residence				0.023	0.878
Rural	23	10 (25.00)	13 (23.64)		
Urban	72	30 (75.00)	42 (76.36)		

**Table 2:** Effect of early multidisciplinary collaboration on complications in critically ill patients (*n* (%)).

Categories	Control group (*n* = 40)	Research group (*n* = 55)	*χ* ^2^	*P*
ICU-AW	16 (40.00)	2 (3.64)	19.940	<0.001
Deep vein thrombosis	4 (10.00)	0 (0.00)	5.742	0.017
Pressure sores	3 (7.50)	0 (0.00)	4.260	0.039
Ventilator-associated pneumonia	6 (15.00)	2 (3.64)	3.878	0.049

## Data Availability

The labeled datasets used to support the findings of this study are available from the corresponding author upon request.
